# Targeting the gut-lung axis by synbiotic feeding to infants in a randomized controlled trial

**DOI:** 10.1186/s12915-023-01531-3

**Published:** 2023-02-20

**Authors:** Kotryna Simonyté Sjödin, Andreas Sjödin, Marek Ruszczyński, Mette Bach Kristensen, Olle Hernell, Hania Szajewska, Christina E. West

**Affiliations:** 1 Department of Clinical Sciences, Pediatrics, Umeå University, Umeå, 901 85 Sweden; 2 Division of CBRN Security and Defense, FOI – Swedish Defense Research Agency, Umeå, Sweden; 3 Department of Paediatrics, The Medical University of Warsaw, Warsaw, Poland; 4 Arla Foods, amba, Aarhus, Denmark

**Keywords:** Antibiotics, Bifidobacteria, Infant gut microbiota, Prebiotics, Probiotics, *Klebsiella*, Lower respiratory tract infection

## Abstract

**Background:**

Formula-fed infants are at increased risk of infections. Due to the cross-talk between the mucosal systems of the gastrointestinal and respiratory tracts, adding synbiotics (prebiotics and probiotics) to infant formula may prevent infections even at distant sites. Infants that were born full term and weaned from breast milk were randomized to prebiotic formula (fructo- and galactooligosaccharides) or the same prebiotic formula with *Lactobacillus paracasei* ssp. *paracasei* F19 (synbiotics) from 1 to 6 months of age. The objective was to examine the synbiotic effects on gut microbiota development.

**Results:**

Fecal samples collected at ages 1, 4, 6, and 12 months were analyzed using 16S rRNA gene sequencing and a combination of untargeted gas chromatography-mass spectrometry/liquid chromatography-mass spectrometry. These analyses revealed that the synbiotic group had a lower abundance of *Klebsiella*, a higher abundance of *Bifidobacterium breve* compared to the prebiotic group, and increases in the anti-microbial metabolite d-3-phenyllactic acid. We also analyzed the fecal metagenome and antibiotic resistome in the 11 infants that had been diagnosed with lower respiratory tract infection (cases) and 11 matched controls using deep metagenomic sequencing. Cases with lower respiratory tract infection had a higher abundance of *Klebsiella* species and antimicrobial resistance genes related to *Klebsiella pneumoniae*, compared to controls. The results obtained using 16S rRNA gene amplicon and metagenomic sequencing were confirmed in silico by successful recovery of the metagenome-assembled genomes of the bacteria of interest.

**Conclusions:**

This study demonstrates the additional benefit of feeding specific synbiotics to formula-fed infants over prebiotics only. Synbiotic feeding led to the underrepresentation of *Klebsiella*, enrichment of bifidobacteria, and increases in microbial degradation metabolites implicated in immune signaling and in the gut-lung and gut-skin axes. Our findings support future clinical evaluation of synbiotic formula in the prevention of infections and associated antibiotic treatment as a primary outcome when breastfeeding is not feasible.

**Trial registration:**

ClinicalTrials.gov NCT01625273. Retrospectively registered on 21 June 2012.

**Supplementary Information:**

The online version contains supplementary material available at 10.1186/s12915-023-01531-3.

## Background

Infants are at increased risk of infections, and respiratory tract infections are a leading cause of morbidity and mortality globally [1]. Although the respiratory and gastrointestinal tracts are separate, they share a mucosal immune system called the “gut-lung axis.” Within the “gut-lung axis” there is a cross-talk between the gut and lung in a two-way manner, including immune and microbial interactions due to the systemic circulation of bacterial ligands, bacterial metabolites, and migrating immune cells [2]. The majority of respiratory tract infections in infancy are confined to the upper respiratory tract, but around one-third of the infected infants will develop a more severe course with symptoms from the lower respiratory tract [1]. Bacterial coinfection in viral lower respiratory infection (LRTI) [1] is common and requires antibiotic treatment, which may negatively impact the developing gut microbiome [3–5]. The establishment of the gut microbiome in the first years of life is shaped by the interactions between the environment, early nutrition, and host-related and microbe-associated factors [6] and parallels the maturation of mucosal and systemic immune responses [7–10].

Breastfeeding is the gold standard in infant nutrition and reduces the frequency of episodes of LRTI [11], otitis media [12], and gastrointestinal infections [11]. There is now emerging interest in feeding infants formula with added pre- and probiotics, termed synbiotics [13, 14], when breastfeeding is not feasible. The benefit of breastfeeding on infections is conferred by numerous bioactive components including human milk oligosaccharides (HMOs), present in large quantities in human milk. HMOs are complex and structurally diverse non-digestible oligosaccharides that stimulate the growth of bifidobacteria [15] while formula feeding leads to a more diverse gut microbiota [16]. With human milk as a model, galactooligosaccharides (GOS) and/or fructooligosaccharides (FOS) are added as prebiotics to infant formula in an effort to mimic the effects of HMOs, although they are not as complex and structurally diverse as HMOs. In addition to the enrichment of bifidobacteria, breastfeeding promotes colonization with *Lactobacillus casei*, *L.casei*/*paracasei*, and *L. johnsonii*/*L. gasseri* [16], also added as probiotics to infant foods. Synbiotics have been defined as “a mixture comprising live microorganisms and substrate(s) selectively utilized by host microorganisms that confers a health benefit on the host” [13], and there is evidence that they stimulate the developing immune system [6, 17]. Although comparative studies are few [18], synbiotics are theorized to have more global effects on infant gut microbiota composition and functions than prebiotics alone. In line with the concept of a “gut-lung axis,” emerging research shows that synbiotics prevent infections even at distant sites, and in a randomized controlled trial (RCT), synbiotics prevented both sepsis and LRTI in preterm infants [19].

In an RCT, we compared the effects of feeding healthy, term infants formula with added prebiotics (FOS and GOS) versus synbiotics (FOS, GOS, and probiotic *Lactobacillus paracasei* ssp. *paracasei* strain F19) on growth and adverse events [20]. The genus *Lactobacillus* was recently reclassified, and *Lactobacillus paracasei* is now classified as *Lacticaseibacillus paracasei* [21]; however, for consistency with our previous paper reporting results from this RCT [20], we use the old classification. Exclusively formula-fed infants were randomized to intake of standard formula with prebiotics or synbiotics from 1 to 6 months and followed until 12 months of age. As judged by adequate growth and lack of adverse events, the synbiotic formula was safe [20]. Although the RCT was not primarily designed for the prevention of infections, there was a reduction in the cumulative incidence of a physician-diagnosed LRTI in the synbiotic compared with the prebiotic group [20].

**Fig. 1:**
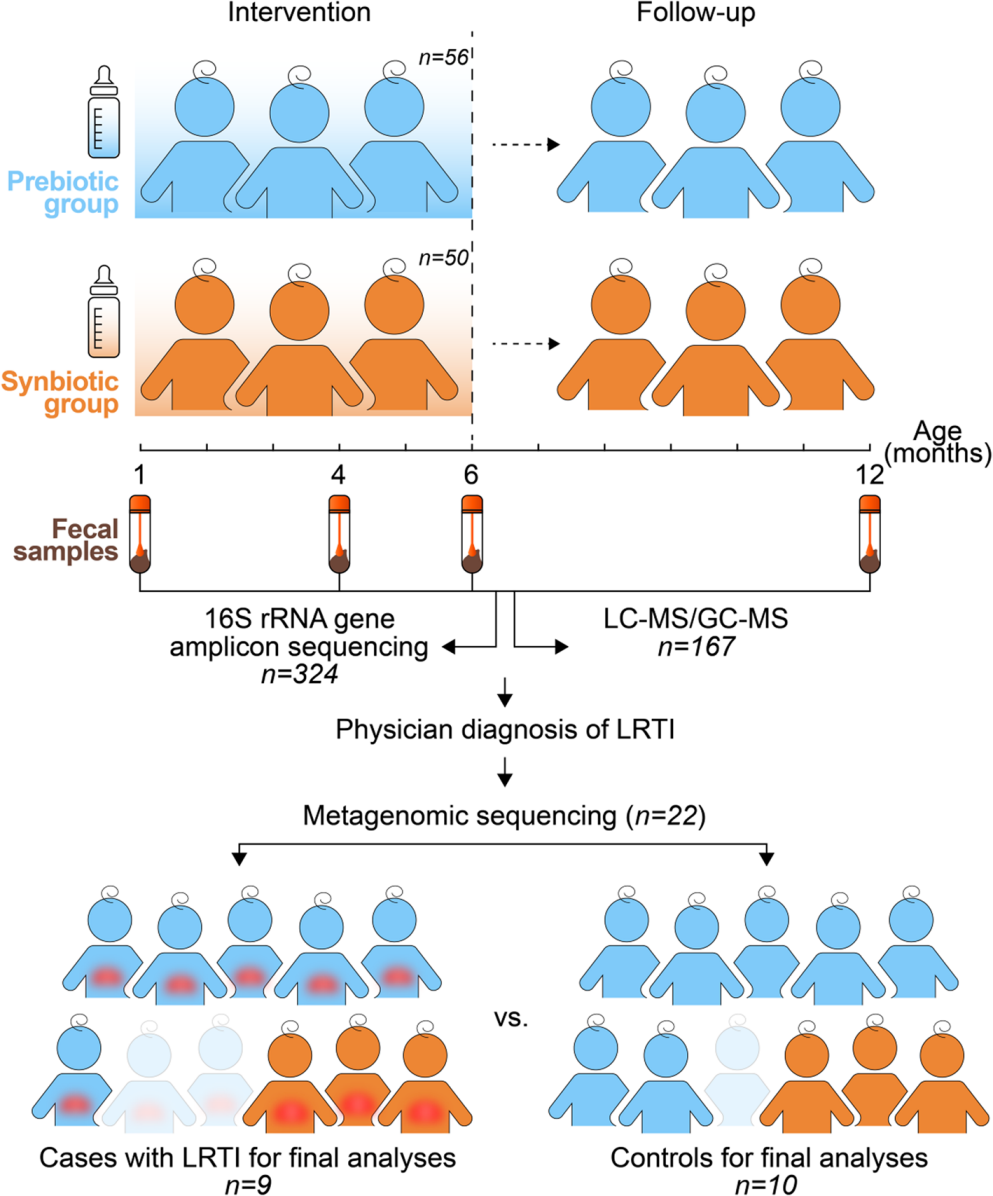
Overview of study design. Of the 106 included infants, 56 were fed formula with prebiotics (prebiotic group; blue), and 50 were fed the same prebiotic formula with *Lactobacillus paracasei* ssp. *paracasei* F19 (synbiotic group; orange) from 1 to 6 months of age. Follow-up continued until 12 months of age. We used 16S rRNA gene amplicon sequencing of 324 fecal samples collected at ages 1, 4, 6, and 12 months for the analysis of gut microbial composition. The fecal metabolome at these ages was investigated in 54 infants (193 samples) using a combination of untargeted gas chromatography-mass spectrometry/liquid chromatography-mass spectrometry (GC–MS/LC–MS). Eleven infants were diagnosed with lower respiratory tract infection (LRTI) by a physician in the first year of life. These and eleven matched controls were selected for deep metagenomic sequencing. In the final analyses, the metagenome and antibiotic resistome in fecal samples of 9 infants with LRTI and 10 matched controls were examined

The objective of this study was to examine the effects of synbiotics on longitudinal infant gut microbiota development in 106 infants using 16S rRNA gene amplicon sequencing of 324 fecal samples collected at ages 1, 4, 6, and 12 months. At these ages, we also analyzed the fecal metabolome as a functional readout of the synbiotic intervention in 54 infants (193 samples) using a combination of untargeted gas chromatography-mass spectrometry/liquid chromatography-mass spectrometry (GC–MS/LC–MS). Using deep metagenomic sequencing, we took the opportunity to examine the metagenome and antibiotic resistome in fecal samples of 11 infants with LRTI and 11 matched controls (Fig. 1).

Here, we demonstrate the compositional and functional changes of infant gut microbiota in relation to synbiotic compared to prebiotic feeding and antibiotic-treated LRTI.

## Results

### Characteristics of the study population

From the initial study [20], we included all infants with available fecal samples for 16S rRNA gene sequencing (Fig. 1). A description of their characteristics is presented in Table 1. Of these, 54 infants contributed at least three samples and were selected for fecal metabolic profiling (Fig. 1). There were no differences in baseline demographic characteristics, number of infants assigned to receive prebiotic or synbiotic formula, nor anthropometric data at any study visit (Table 1). At 4 and 6 months of age, there was a higher frequency of physician-diagnosed LRTI and use of antibiotics in the prebiotic group although the difference was not statistically significant (*p* = 0.19, test of equal proportions). The 11 infants with physician-diagnosed LRTI (cases) and 11 controls matched for age, sex, and intervention (Fig. 1, Table 2) were included for the analysis of the metagenome and antimicrobial resistome. All infants with LRTI had been prescribed antibiotics (amoxicillin/clavulanic acid, ampicillin, or cefuroxime) for a duration of 7 to 10 days. The fecal sampling time was within 30 days after antibiotic treatment. After metagenomic sequencing, we obtained additional clinical information on three of these infants (two cases and one control). In addition to the LRTI diagnosis, they had comorbidities and multiple medications at the time of sampling, which is why we excluded these from further analyses. Consequently, 9 infants with LRTI and 10 controls were included in the final analyses (Fig. 1).

**Table 1:** Study population

	Visit 1* (1 month of age)		Visit 2* (4 months of age)		Visit 3* (6 months of age)		Visit 4* (12 months of age)	
	**Prebiotics**	**Synbiotics**	**Prebiotics**	**Synbiotics**	**Prebiotics**	**Synbiotics**	**Prebiotics**	**Synbiotics**
***N* (**^a^**)**	56	50	47	42	38	33	31	33
**Girls, %**	43	56	45	50	47	52	52	52
**Siblings**	34	35	na	na	na	na	na	na
**Gestational age, weeks**	39 ± 1.07	40 ± 1.2	na	na	na	na	na	na
**Age, months**	0.7 ± 0.2	0.8 ± 0.2	3.9 ± 0.3	4.0 ± 0.3	5.9 ± 0.4	6.0 ± 0.2	12.1 ± 0.4	12.1 ± 0.3
** Length, cm**	55 ± 2.6	55.6 ± 2.5	65.2 ± 2.6	66.4 ± 3.2	69.9 ± 3.2	70.5 ± 3.7	81.2 ± 3.9	79.7 ± 5.1
**Weight, kg**	4.0 ± 0.5	4.1 ± 0.5	6.9 ± 0.9	7.1 ± 0.8	8.1 ± 0.8	8.3 ± 0.8	10.2 ± 1.1	10.1 ± 0.8
**Head circumference, cm**	36.5 ± 1.9	36.7 ± 1.9	41.5 ± 1.4	41.5 ± 1.3	43.3 ± 1.3	43.2 ± 1.1	46.2 ± 2.0	46.1 ± 1.3
**BMI *z*-score**	− 0.7 ± 0.9	− 0.6 ± 0.9	− 0.4 ± 1.0	− 0.6 ± 1.2	− 0.5 ± 1.1	− 0.4 ± 1.4	− 1.0 ± 1.5	− 0.6 ± 1.5
**LRTI**	na	na	3	1	3	0	2	2
**Antibiotic use**	na	na	4	1	3	2	3	2

*LRTI* Lower respiratory tract infection

^a^All subjects were vaginally delivered and predominantly formula-fed at inclusion

^*^p > 0.05 data are presented as mean ± SD; the Mann–Whitney *U* test or test of equal or given proportions was used to assess the differences between the groups

**Table 2:** Characteristics of infants with diagnosed lower respiratory tract infections (cases) and their matched controls

	Cases (*n* = 11)*	**Controls (*n* = 11)**
**Intervention, prebiotics/synbiotics**	8/3	8/3
**Girls**	6	6
**Age, months**	7.2 ± 3.6	7.4 ± 3.6
** Length, cm**	72.0 ± 8.1	72.7 ± 8.2
**Weight, kg**	8.5 ± 1.5	8.5 ± 1.5
**Head circumference, cm**	43.1 ± 2.4	43.9 ± 2.6
**BMI *z*-score**	− 0.5 ± 1.3	− 0.7 ± 1.3
**LRTI**	11	0
**Antibiotic use**	11	0

*LRTI* Lower respiratory tract infection

^*^p > 0.05 data are presented as mean ± SD; the Mann–Whitney *U* test or test of equal or given proportions was used to assess the differences between the groups

### Synbiotic effects on gut microbial composition

**Fig. 2:**
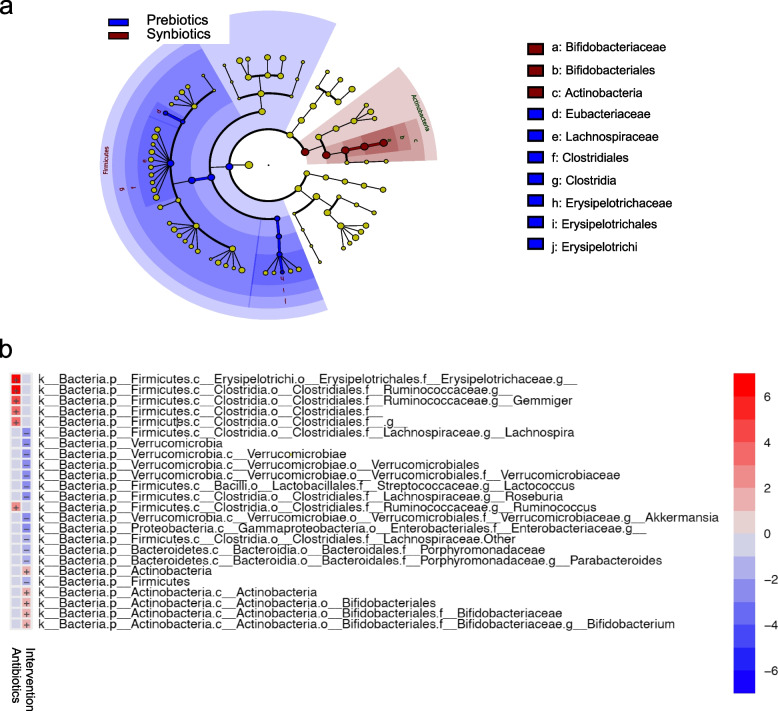
Discriminant taxa between the prebiotic and synbiotic groups at 6 months of age. **a** A cladogram presenting a significantly higher relative abundance of *Bifidobacteriaceae* in the synbiotic group (orange) and a higher relative abundance of *Erysipelotrichaceae*, *Lachnospiraceae*, and *Eubacteriaceae* in the prebiotic group (blue) at age 6 months, i.e., 5 months after the start of the intervention. LEfSe was used to investigate the discriminant taxa, and the LEfSe algorithm uses the Kruskal–Wallis sum rank test, unpaired Wilcoxon rank-sum test, and LDA, where *α* = 0.05 and LDA threshold = 2.0. **b** A heatmap presenting that multivariable analysis using MaAsLin2 identified that *Bifidobacteriaceae* correlated with the intervention (synbiotic feeding) (*p* = 0.02) and that *Erysipelotrichaceae* were strongly associated with antibiotic treatment (*p* < 0.001) (Benjamini-Hochberg, *q*-values were *q* = 0.2 for *Bifidobacteriaceae* and *q* = 0.0009 for *Erysipelotrichaceae*)

First, we applied the linear discriminant effect size (LEfSe) [22] method to identify the discriminative taxa according to the intervention at 6 months of age, i.e., when the infants had consumed the study formulas for 5 months and the putative effect of the intervention would be most evident. This analysis determined that four taxa best discriminated the groups at that age; *Bifidobacteriaceae* were overrepresented whereas *Eubacteriaceae*, *Lachnospiraceae*, and *Erysipelotrichaceae* were underrepresented in the synbiotic (*n* = 33) compared with the prebiotic (*n* = 38) group (Fig. 2a). We then performed multivariable analysis by linear models analysis using MaAsLin2 [23] to identify if the taxonomical differences were only intervention dependent or if treatment with antibiotics contributed to these differences. As presented in the heatmap (Fig. 2b), *Bifidobacteriaceae* were associated with the intervention (synbiotic feeding) (*p* = 0.02), and *Erysipelotrichaceae* were strongly associated (*p* < 0.001) with antibiotic treatment (Benjamini-Hochberg, *q*-values were *q* = 0.2 for *Bifidobacteriaceae* and *q* = 0.0009 for *Erysipelotrichaceae*).

The longitudinal design made it possible to study the gut microbial compositional changes according to both intervention and age. We applied the ANCOM BC [24] pipeline to investigate the microbial differential abundance at the genus (D_5 in SILVA’s taxonomy tree) and species (D_6 in SILVA’s taxonomy tree) levels, longitudinally between the prebiotic and synbiotic groups of all fecal samples (*n* = 324) included for 16S rRNA gene amplicon sequencing, normalized for baseline, i.e., at 1 month of age. The synbiotic group had a lower abundance of *Klebsiella* (*p* = 0.01) but a higher abundance of *Bifidobacterium breve* compared to the prebiotic group (*p* = 0.002).

### Synbiotic effects on gut microbiota diversity and maturation measures

We assessed α-diversity, here presented as Faith’s PD (Additional file 1: Fig. S1a), which is a qualitative measure of community richness incorporating a phylogenetic relationship. As expected, α-diversity increased during the first year of life in both intervention groups. The α-diversity at 4 months of age was lower in the synbiotic (*n* = 42) compared with the prebiotic group (*n* = 47) and at 12 months (*n* = 33 in the synbiotic and *n* = 31 in the prebiotic group) (*p* < 0.01, Kruskal–Wallis) (Additional file 1: Fig. S1a). We also investigated the microbial dissimilarity (β-diversity) between the intervention groups over time based on unweighted UniFrac distance matrices among all fecal samples (*n* = 324) (Additional file 1: Fig. S1b). The overall difference was significant (*p* < 0.001 for all comparisons, PERMANOVA, number of permutations 999). To evaluate the microbial maturation according to the intervention, we calculated the microbiota-by-age *Z* (MAZ) score [25, 26] and observed no significant differences between the groups (Additional file 1: Fig. S1c).

### Synbiotic effects on gut microbial metabolic activity

**Fig. 3:**
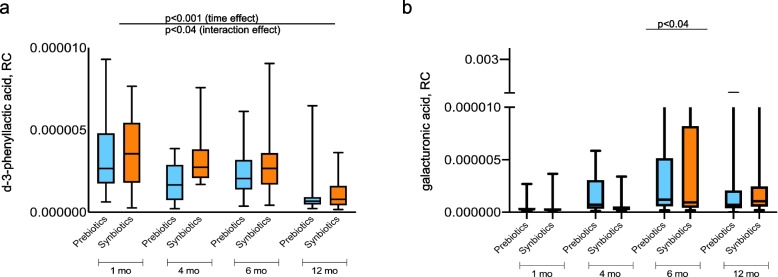
Metabolites derived from microbial fermentation of phenylalanine and pectins were increased in the synbiotic group. **a** Box plots presenting the antimicrobial metabolite d-3-phenyllactic acid (PLA), which is derived from the microbial fermentation of phenylalanine in the prebiotic (blue) compared with the synbiotic (orange) group, using a mixed-effects model, *p* < 0.001 for time and p = 0.04 for interaction. **b** Box plots presenting the galacturonic acid, which is derived following microbial degradation of pectins. Galacturonic acid was increased in the synbiotic group (orange) at age 6 months (Mann–Whitney *U* test, *p* = 0.039). Data are presented as mean ± SD. Prebiotic group (*n* = 23 at 1 and 4 months, *n* = 27 at 6 months, and *n* = 26 at 12 months). Synbiotic group (*n* = 22 at 1 month, *n* = 25 at 4 months, *n* = 20 at 6 months, and *n* = 27 at 12 months). RC-relative concentration

Next, we investigated the metabolome as a functional readout of gut microbial metabolic activity (Fig. 1 and Additional file 1: Table S1 displaying characteristics of this sub-cohort). Here, we took the same approach as for the 16S rRNA gene sequencing data by analyzing the metabolite changes from baseline at age 1 to 6 months post-intervention, i.e., at age 12 months. This revealed the differences in unique metabolites according to the intervention. The antimicrobial metabolite d-3-phenyllactic acid (PLA), which is derived from the metabolism of phenylalanine by taxa, e.g., lactic acid bacteria (LAB) [27], was increased over time (*p* < 0.001 and *p* = 0.04 for time and interaction, respectively, mixed-effects model) in the synbiotic compared with the prebiotic group (Fig. 3a). As for the 16S rRNA gene sequencing data, we investigated the putative changes in metabolites at 6 months of age as this represented the last sampling during the intervention where we expected the intervention effect to be greatest. At that age, galacturonic acid, which is derived following microbial degradation of pectins, was increased (*p* = 0.039, Mann–Whitney *U* test) in the synbiotic (*n* = 20) compared with the prebiotic group (*n* = 27) (Fig. 3b).

**Fig. 4:**
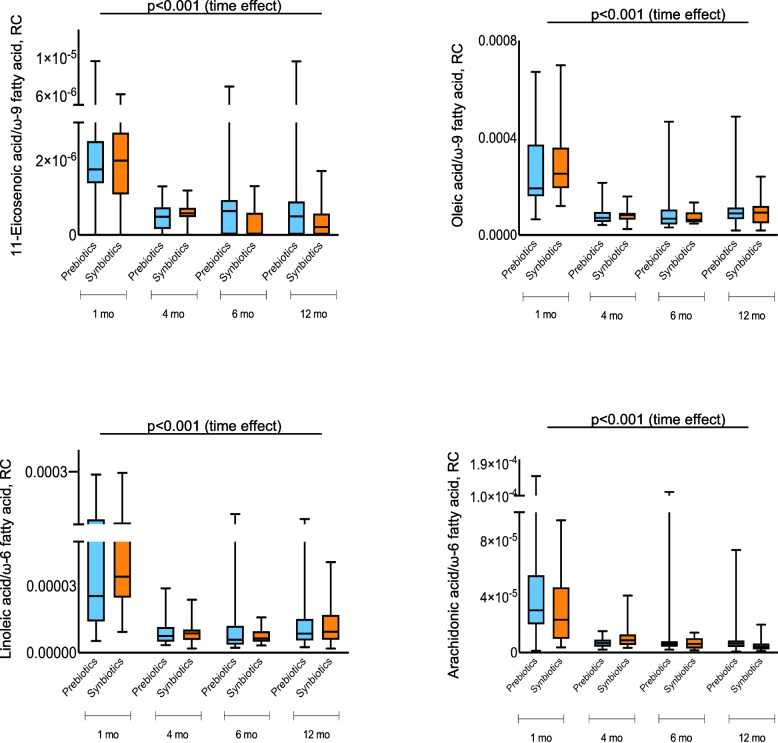
The decrease in unsaturated fatty acids is age-related. Box plots presenting mono- and polyunsaturated acids in the prebiotic (blue) and synbiotic (orange) groups using a mixed-effects model, *p* < 0.001 for time. Data are presented as mean ± SD. Prebiotic group (*n* = 23 at 1 and 4 months, n = 27 at 6 months, and *n* = 26 at 12 months). Synbiotic group (*n* = 22 at 1 month, *n* = 25 at 4 months, *n* = 20 at 6 months, and *n* = 27 at 12 months). RC, relative concentration

Analysis of the 193 samples (Fig. 1) revealed that the overall metabolite profile at 12 months differed significantly from the earlier ages (*p* < 0.001, paired Kruskal–Wallis test, Additional file 1: Fig. S2a) but not according to the intervention (Additional file 1: Fig. S2b). Fatty acid metabolic pathway-related fecal metabolites, i.e., mono- and polyunsaturated fatty acids, were also significantly related to age (*p* < 0.0001, mixed-effects model) but not to the intervention (Fig. 4). At 1 month (baseline), the relative concentrations of oleic, arachidonic, linoleic, and eicosenoic acids in feces were significantly higher compared to the later ages, likely reflecting the fatty acids present in breast milk and formula consumed before the intervention started, and then remained low throughout the first year of life.

### Enrichment of *Klebsiella* species in antibiotic-treated LRTI

To further explore the connection between gut microbiota and LRTI, we shotgun-sequenced fecal samples from the infants with LRTI (cases) and their matched controls (Fig. 1, Table 2). With metagenomics, we detected four kingdoms with an absolute dominance of Bacteria (99%). The corresponding numbers for Virus were 0.14%, Eukaryota 0.05%, and Archaea 2.9e^−05^%, with high individual variability according to health status and age. The small sample size (9 cases and 10 controls) and the high interindividual variation limit the statistical analyses of metagenomic data.

First, we focused on Actinobacteria, the dominant phylum in both cases and controls during the first 6 months of life. Metagenomic sequence reads were classified into 35 *Bifidobacterium* species (Additional file 1: Fig. S3) with the highest relative abundance of *B. longum*, *B. breve*, *B. adolescentis*, and *B. bifidum*. Adult-type *B. pseudocatenulatum* was overrepresented in cases with LRTI (*n* = 9) compared with controls (*n* = 10) (*p* = 0.03; Mann–Whitney test). With the exception of *B. pseudocatenulatum* and *B. bifidum*, all other bifidobacterial species were more abundant in controls, although the differences did not reach statistical significance.

Next, we focused on *Klebsiella* species that are part of the commensal microbiota of the nose, mouth, and gut. *K. pneumoniae* and *K. oxytoca*, however, are opportunistic pathogens colonizing the mucosal surfaces, and from the mucosa, they may disseminate to other tissues and cause severe infections, including infections in the lower respiratory tract [28]. *Klebsiella* was overrepresented in cases (*n* = 9) compared to controls (*n* = 10) (*p* = 0.06, Mann–Whitney test). Four species of *Klebsiella*, i.e., *K. pneumoniae*, *K. oxytoca*, *K. grimontii*, and *K. michiganensis*, were dominant in cases (*n* = 9) (*p* = 0.1 for *K. pneumoniae* and *p* = 0.07 for the other *Klebsiella* species; Mann–Whitney test, Additional file 1: Fig. S4) compared to controls (*n* = 10), thus suggestive as a causative or contributing pathogen in LRTI.

### Higher relative number of sequence reads related to *Klebsiella pneumoniae* antimicrobial resistance genes in LRTI

**Fig. 5:**
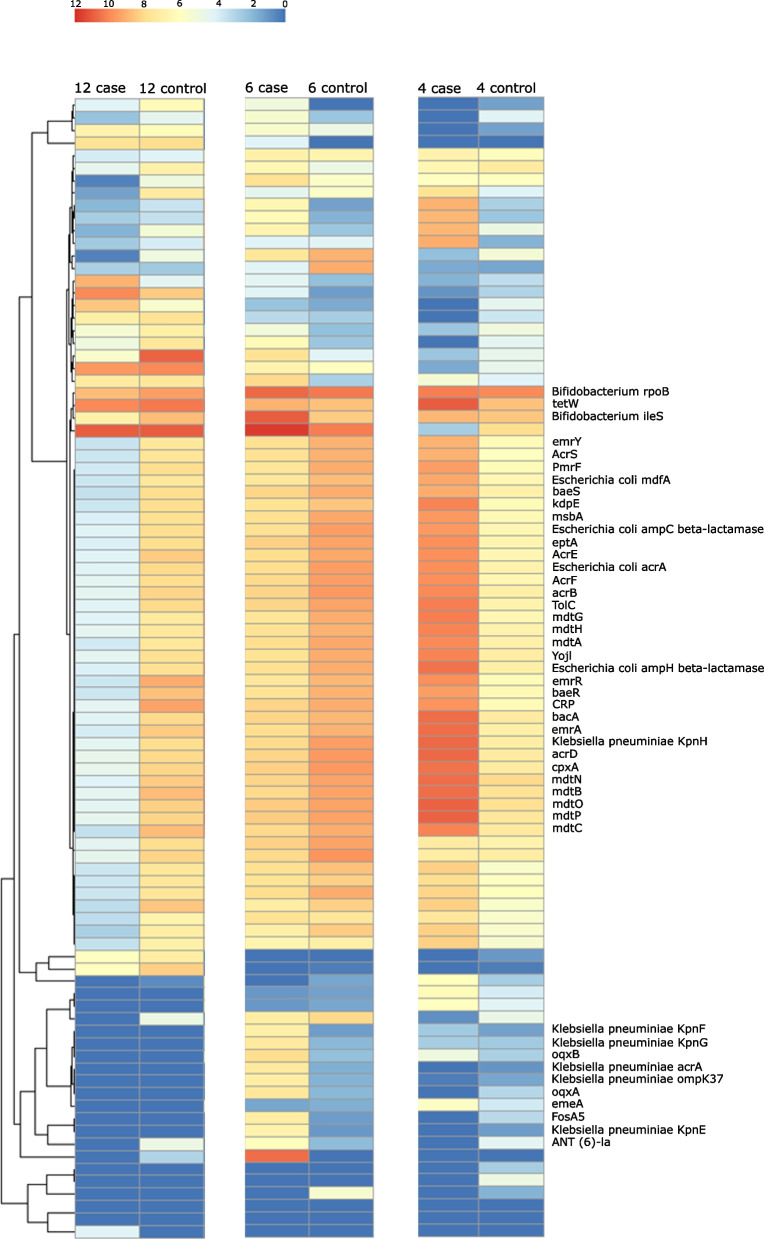
Differential abundance of genes associated with antimicrobial resistance in infants with diagnosed lower respiratory tract infection compared with matched controls. Genes related to proteins involved in antibiotic efflux systems in the bacterial species related to gut dysbiosis, i.e., *E. coli*, *Shigella* ssp., *Enterococcus* ssp., *Salmonella* ssp., *C. difficile*, are more abundant in cases with LRTI compared to healthy controls at 4 months. At age 6 months, cases with LRTI have an increased abundance of genes related to *K. pneumoniae*. LRTI, lower respiratory tract infection

We then characterized the antimicrobial resistome encoded in the infant gut microbiota using metagenomic sequence reads (Fig. 5). According to the Comprehensive Antibiotic Resistance Database (CARD) [29], the relative number of sequence reads related to *Klebsiella pneumoniae* antimicrobial (AMR) genes (*KpnE*, *KpnF*, *KpnG*, *oqxA*, *oqxB*, *KacrA*, *OmpK37*, *FosA5*, and *ANT(6)-Ia*) was higher in cases (*n* = 9), suggesting an infection-related dysbiosis in cases compared to controls (*n* = 10) at age 6 months. The relative number of sequence reads related to AMR genes encoding for proteins involved in antibiotic efflux system (*emrY*, *AcrS*, *E. coli mdfA*, *baeS*, *kdpE*, *msbA*, *E.coli ampC beta-lactamase*, *eptA*, *AcrF*, *acrB*, *TolC*, *mdtG*, *mdtH*, *mdtA*, *Yojl*, *E.coli ampH beta-lactamase*, *emrR*, *baeR*, *CRP*, *bacA*, *emrA*, *K.pneumoniae KpnH*, *acrD*, *cpxA*, *mdtN*, *mdtB*, *mdtO*, *mdtP*, *mdtC*) in bacterial species related to gut dysbiosis, e.g., *E. coli*, *Shigella* ssp., *Enterococcus* ssp., *Salmonella* ssp., and *C. difficile*, was higher in cases (*n* = 9) compared to controls (*n* = 10) during the intervention. That pattern was reversed at 12 months of age.

### Cultivation-independent recovery of genomes from high-quality metagenomes assigns AMR genes to bacterial species

**Fig. 6:**
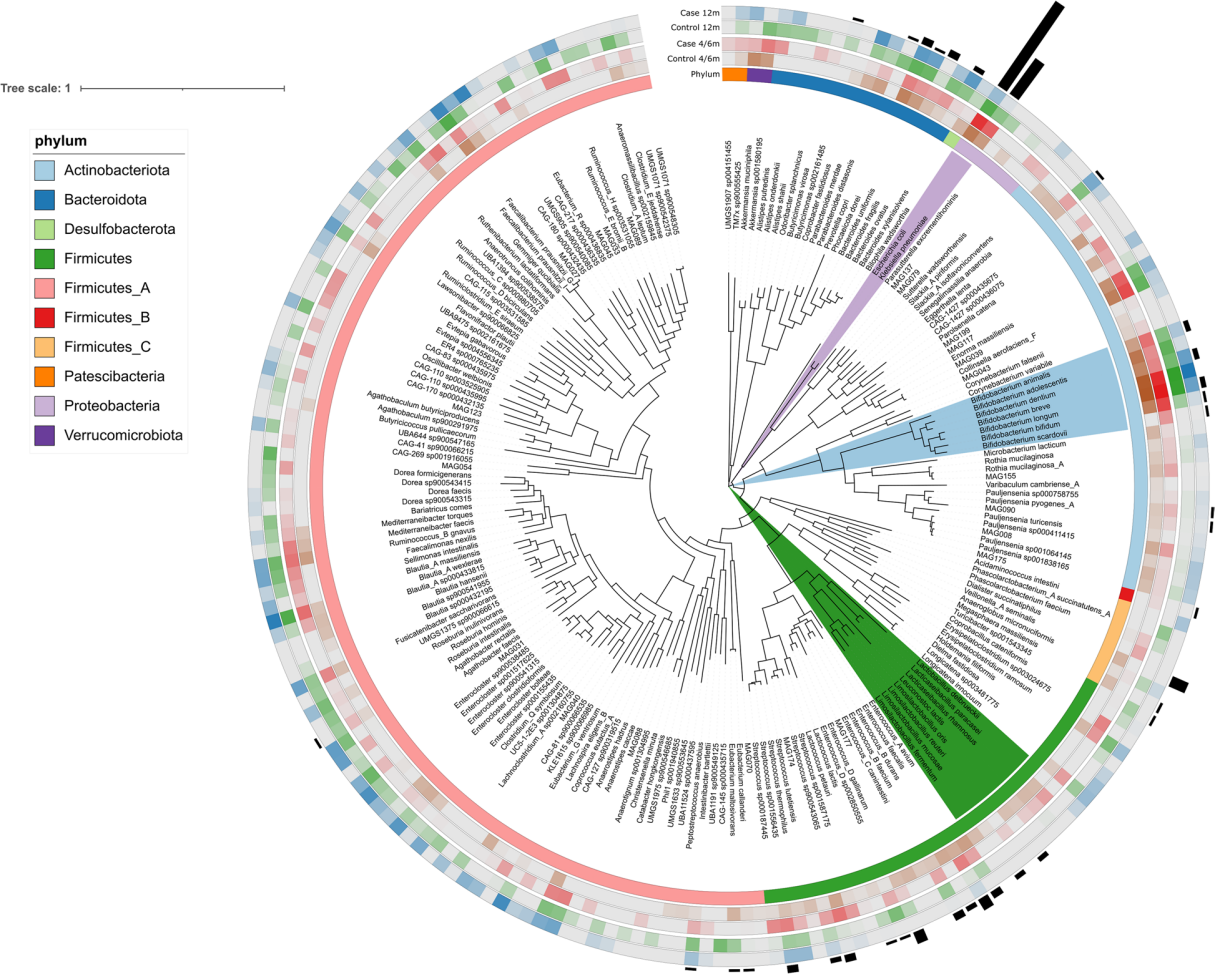
Phylogenetic tree of cultivation-independently recovered MAGs. Two hundred three recovered high-quality MAGs from shotgun sequences that were characterized and placed in a phylogenetic tree. The outer rings provide information on taxonomy, LRTI-related metadata, and AMR genes. From inside out: the first circle color codes 10 different MAGs phylum; the 2–4 circles present an average abundance of different MAGs in a certain group at a certain time point, i.e., brown: controls at 4 and 6 months (combined), red: LRTI cases at 4 and 6 months, green: controls at 12 months, and blue: LRTI cases at 12 months. In the top layer of the figure, the black bars present the number of AMRs found in certain MAG. MAGs, metagenome-assembled genomes; AMR, antimicrobial resistance genes; LRTI, lower respiratory tract infection

We were further interested in better understanding our observations and assigning them to bacterial species. Recent advances in sequencing throughput and computational techniques allow metagenome-assembled genomes (MAGs) to be recovered from high-diversity environments [30]. Here, we report 203 recovered high-quality MAGs covering 10 phyla according to GTDB taxonomy [31] and visualized as a phylogenetic tree (Fig. 6). We confirm the results obtained using 16S rRNA gene amplicon and read-based analysis of shotgun sequencing by successful recovery of the MAGs of bacteria of interest, i.e., *E. coli*, *K. pneumoniae*, *B. animalis*, *B. adolescentis*, *B.dentinum*, *B. breve*, *B. longum*, *B. bifidum*, *B. scardovii*, *L. delbrueckii*, *Lacticaseibacillus paracasei* (previously known as *Lactobacillus paracasei*) [21], *L. rhamnosus*, *Leuconostoc lactis*, *Limosilactobacillus oris* (previously known as *Lactobacillus oris*) [21], *L. reuteri*, *L. mucosae*, and *L. fermentum*. The lines around the phylogenetic tree in Fig. 6 present and reflect the average abundance of the recovered MAGs between cases and controls at a certain time point, i.e., 4 and 6 months combined, and at 12 months of age. Additionally, we were able to detect 94 different AMR genes in our MAGs. As expected, the highest abundances of AMR genes, corresponding to 23 genes, were detected in *E. coli* and *K. pneumoniae*. We were able to confirm the subgroup of the 2 AMR genes related to *Bifidobacterium*. The rest of the genes are spread over the phylogenetic tree, however, mainly associated to the genera within *Enterococcus* and *Streptococcus*.

## Discussion

This RCT compared feeding prebiotic infant formula with the same prebiotic infant formula supplemented with probiotic *Lactobacillus* F19 (synbiotics) until 6 months of age on infant gut microbiota development in the first year of life. Four taxa discriminated the intervention groups at age 6 months when the infants had consumed the study formulas for 5 months; *Bifidobacteriaceae* were overrepresented whereas *Eubacteriaceae*, *Lachnospiraceae*, and *Erysipelotrichaceae* were underrepresented in the synbiotic compared with the prebiotic group. The multivariable analysis further identified that synbiotic feeding for 5 months was associated with *Bifidobacteriaceae* whereas antibiotic treatment was associated with *Erysipelotrichaceae*. This is consistent with previous studies that reported *Erysipelotrichaceae* to flourish following antibiotic treatment [32] and that a single course of amoxicillin in infancy disrupted normal microbiota development by abruptly reducing bifidobacteria abundance followed by replacement with clostridia and enterobacteria [5]. *Eubacteriaceae* and *Lachnospiraceae* increased in an age-dependent manner from 9 to 18 months in a Danish cohort [33], and the underrepresentation of these taxa in the prebiotic group could thus reflect a less infant-like microbiota at 6 months of age.

One of the novelties of this study is the follow-up in the first year of life, i.e., until 6 months post-intervention. At 12 months of age, α-diversity was lower in the synbiotic group, which is consistent with a previous study that reported a slower increment in α-diversity in cow’s milk allergic infants fed another synbiotic formula compared with the same formula without synbiotics [34]. Longitudinal analysis in the first year of life further identified reduced *Klebsiella* abundance in the synbiotic group but no other differences at the genus level according to the intervention. At 4 and 6 months of age, *B. breve*, a species commonly found in the gut of breastfed infants [16], was enriched in the synbiotic compared with the prebiotic group. As the resolution at the species level using 16S rRNA gene sequencing is uncertain [35] the results should be interpreted with caution. Verification with metagenomic sequencing, which could not be undertaken since we analyzed a subsample and not all samples, should be applied in future studies.

The advantage of applying GC–MS/LC–MS as a functional readout of the synbiotic intervention was clearly shown as it detected increases in PLA, a by-product of phenylalanine metabolism in LAB [36]. Previous reports have demonstrated LAB metabolites e.g. short-chain fatty acids (SCFAs) to act as a link between the microbiota and the host immune system by activation of G protein-coupled receptors (GPCRs), which are expressed in intestinal epithelial cells and immune cells in the gut mucosa [37, 38]. Still, the mechanisms behind the beneficial effects of LAB on host physiology are limited, and it was not until very recently that PLA was demonstrated to signal through a specific GPCR for hydroxycarboxylic acids (HCAR) in humans, namely HCAR_3_ [39]. Our observation that synbiotic feeding increases PLA, although slightly, could be of high clinical relevance since HCAR_3_ are expressed in various immune cells as well as the lung and skin. Signaling via the HCAR_3_ may thus be implicated in the gut-lung [2] and gut-skin [40] axes. PLA also has anti-microbial properties and inhibits the growth of Gram-negative bacteria, e.g., *Klebsiella* as well as Gram-positive *Enterococcus* and *Staphylococcus* in vitro [41].

Gut microbial composition and functions develop as complementary foods are introduced [16, 33, 42]. Pectins are found in fruits and vegetables, are added to yoghurts, and thus form a part of complementary foods introduced around 6 months of age. Pectins are a family of complex polysaccharides with an α(1,4)-linked d-galacturonic acid or rhamnogalacturonan backbone. While human enzymes cannot digest pectins, they can be degraded by taxa, e.g., *Lactobacillus*, *Bifidobacterium*, and *Bacteroides* [43, 44]. At age 6 months, there were increases in galacturonic acid in the synbiotic group, but since we did not collect detailed dietary data, it remains undecided if this increase is due to the intervention or differences in intake of foods rich in pectins between the groups.

Deep metagenomic sequencing further revealed that *Klebsiella* species were overrepresented in antibiotic-treated infants with LRTI compared with antibiotic-naïve controls. *Klebsiella* is of high concern in the context of antimicrobial resistance [28]. In accordance with the overrepresentation of *Klebsiella* species, the relative number of sequence reads related to *Klebsiella pneumoniae* AMR genes associated with antibiotic efflux and its regulation was higher in antibiotic-treated infants with LRTI at 6 months of age. At age 4 months, there was also a relatively higher number of sequence reads related to AMR genes encoding for proteins involved in antibiotic efflux system in bacterial species related to gut dysbiosis, e.g., *E. coli*, *Shigella* ssp., *Enterococcus* ssp., *Salmonella* ssp., and *C. difficile* in antibiotic-treated infants with LRTI, although this was not seen at later ages. Finally, we used a new culture-independent bioinformatic technique to confirm our findings by generating high-quality MAGs in high-diversity samples. Obtaining genomes from metagenomes is an emerging approach with the potential for large-scale recovery of near-complete genomes [30].

The main strengths of our study are the randomized, controlled prospective design, repeated samplings, and the combined approach of 16S rRNA gene amplicon sequencing and metabolomics, thereby examining the synbiotic effects on both compositional and functional aspects of the gut microbiota. We also consolidated in silico the results obtained using 16S rRNA amplicon and shotgun sequencing by successful recovery of the MAGs of bacteria of interest. There are also limitations; we did not have fecal samples from all participants, and therefore, only a subset was available for gut microbiota analyses. Although commonly used for practical and ethical reasons, analysis of fecal samples may not be representative of changes occurring further up in the intestine. It would also have been valuable to analyze other metabolic aspects, e.g., the SCFA pattern; however, the GC–MS/LC-MC approach did not detect SCFAs, which are highly volatile compounds that require dedicated sample collection, metabolite extraction, and a targeted detection method. We also took the opportunity to characterize the metagenome and antimicrobial resistome in infants with antibiotic-treated LRTI and antibiotic-naïve controls, but these results are preliminary due to the small sample size. Finally, our results are restricted to healthy, term infants and may not be extrapolated to preterm infants nor infants with underlying diseases.

## Conclusions

This RCT demonstrates the additional benefit of feeding specific synbiotics to formula-fed infants over prebiotics only. Synbiotic feeding led to the underrepresentation of *Klebsiella*, enrichment of bifidobacteria, and slight increases in microbial degradation metabolites implicated in immune signaling and in the gut-lung and gut-skin axes. Our results support future clinical evaluation of synbiotic formula in the prevention of infections and associated antibiotic treatment as a primary outcome when breastfeeding is not feasible. This is further underscored by the finding that antibiotic-treated infants with LRTI harbored a higher abundance of *Klebsiella* species and AMR genes encoded in the infant gut microbiome, compared with antibiotic-naïve controls. Ideally, future clinical trials with synbiotics should be coupled with a multi-omics approach to better characterize the compositional and functional changes in the gut microbiome.

## Methods

### Study design

This multicenter, double-blind RCT followed the reporting guidelines of the Consolidated Standards of Reporting Trials (CONSORT), and study details have been published previously [20]. In summary, infants were eligible for recruitment following written informed consent had been obtained from parents. The study was approved by the Ethics Committee of the Medical University of Warsaw and conducted in accordance with Good Clinical Practice and the principles and rules of the Declaration of Helsinki. It was registered at ClinicalTrials.gov (NCT01625273).

### Participants and study settings

As previously described [20], eligible participants were term infants aged ≤ 28 days, vaginally delivered between 38 and 42 weeks of gestation, with a birth weight > 2700 g and < 4200 g, fully weaned from breast milk at age 28 days, and with parents being able to speak and comprehend Polish. The exclusion criteria were any malformation, handicap, or congenital disease that could impact growth; antibiotic treatment; or previous intake of infant formula with pre- and/or probiotics. Four hospitals in Poland (Warsaw, Bydgoszcz, Trzebnica, and Garwolin) or outpatient clinics recruited infants from January 2011 to March 2016. Parents were informed and invited to participate only if infants were not breastfed.

### Interventions and procedures

Eligible infants were randomized to intake of cow’s milk-based infant formula with FOS/GOS (control, prebiotic formula) or identical formula with the addition of *Lactobacillus* F19 (experimental, synbiotic formula) at a dose of 10^9^ colony-forming units per liter of formula. The study formulas were dispensed from inclusion (age 28 days at the latest) until age 6 months (intervention period). The study products’ composition has been published previously [20]. Parents were advised to follow the standard recommendation on the introduction of complementary foods (not before 17 weeks of age and not later than 26 weeks of age) [45]. Study visits took place every 28 days (± 7 days) until age 4 months. Thereafter, study visits took place within 14 days of ages 6, 9, and 12 months. At the study visits, weight, length, and head circumference were measured. Weight (with no clothes on) was measured to the nearest 10 g, and length and head circumference, to the nearest 0.5 cm. WHO Child Growth Standards [http://www.who.int/childgrowth/en/] were used to calculate age-adjusted *z* scores for weight, length, and body mass index (BMI). Health-related data [20] were monitored, and information on physician-diagnosed upper and lower respiratory infections, use of antibiotics, gastrointestinal infections, unscheduled doctor’s visits, hospitalization, and adverse events was collected at each study visit.

### Randomization and blinding

As previously described [20], investigators at the Medical University of Warsaw generated independent allocation sequences and a randomization list (StatsDirect statistical software; StatsDirect Ltd., Altrincham, Cheshire, UK) in blocks of 6 participants. An independent person produced the randomization schedule and monitored all packing and labeling of study products for proper allocation concealment. The study staff, parents, and guardians were uninformed of the group assignments. Randomization codes were covered until data analysis. The study remained blinded to the sponsor, participants, and investigators until the last follow-up, and the statistical analysis was finalized. The blinding was conducted at the production site of the company of the sponsor. Study formulas were delivered to study participants in identical boxes coded in different colors.

### Power analysis

This was a superiority trial, and we estimated that a sample size of 140 (70 in each group) would detect a difference of 0.5 SD in weight at 4 months (primary outcome) with 80% power (5% significance). With an estimated dropout rate of 20%, 90 infants had to be included in each arm. A priori power analyses for the secondary outcomes were not calculated.

### Stool sample collection

Stool samples were collected at home, by spatula into sterile containers at 1, 4, 6, and 12 months of age and stored immediately at home in the freezer. Samples were transported frozen to the research centers both in Poland and Sweden where samples were stored at − 70 °C until analyses.

### Bacterial DNA isolation

Eighty to 160 mg of frozen stool was transferred to Precellys soil grinding SK38 lysing tubes (Bertin Technologies, Montigny-le-Bretonneux, France), and one volume of warm (37 °C) lysis buffer [4% (w/v) SDS, 50 mM TrisHCl pH 8.0, 500 mM NaCl, 50 mM EDTA] was added. Samples were homogenized for 10 min at room temperature, using a Vortex adapter. Lysozyme (Sigma Aldrich Chemie GmbH, Germany; final concentration 6.25 mg/ml) was added and samples were incubated at 37 °C for 30 min, then transferred to 80 °C heating block and incubated for 15 min by inverting every 5 min. The samples were then centrifuged at 4 °C 20,000* g* for 5 min, and supernatants were collected; proteinase K (Roche Diagnostics GmbH, Germany) was added (final concentration 0.4 mg/ml), and the samples were incubated, on a heating block, at 70 °C for 10 min. After incubation, 10 M NH_4_OAc (final concentration 2 M) was added, and samples were incubated on ice for 5 min, then centrifuged at 4 °C 20,000* g* for 10 min. Supernatants were collected, and an equal volume of cold isopropanol was added. The samples were stored on ice for 30 min and thereafter centrifuged at 4 °C 20,000* g* for 20 min. Pellets were washed 2–3 times with cold 70% ethanol, dried, and dissolved overnight in Tris–EDTA buffer (1xTE). The following day, the DNA concentrations were measured using the Qubit dsDNA Broad Range Assay kit (Thermo Fisher Scientific Inc., USA) on Qubit 3.0 fluorometer (Thermo Fisher Scientific Inc., USA). RNAse was added to each sample (final concentration of 1 µg/µl).

### 16S rRNA gene library preparation and sequencing

The sequencing library was prepared according to Earth Microbiome Project’s Protocol [46] with the following modifications; the fused primers were modified to contain barcode sequence on both forward (341F) and reverse (805R) primer and selected to target the V3–V4 regions instead of the V4 region.

The PCR reactions for library preparation were carried out in triplicates as follows: 20 ng of template DNA was mixed with 5PRIME HotMasterMix (Quantabio, USA) consisting of 1U Taq polymerase, 45 nM Cl, 2.5 mM Mg2^+^, 0.2 mM of each dNTP, 0.2 µM of each primer (Eurofins Genomics, Germany), and 0.64 ng bovine serum albumin (BSA) in a final volume of 25 µl per reaction. The PCR conditions were 90 °C for 15 s and 94 °C for 3 min followed by 35 cycles of 94 °C for 45 s, 50 °C for 1 min, and 72 °C for 1.5 min, after which a final elongation step at 72 °C for 10 min was performed. The triplicates were pooled and visualized on 1% agarose gel to estimate the size of the amplicons. DNA concentrations of the amplicons were measured as described in the section above.

Then, libraries were pooled in equimolar concentrations, and the amplicon pool was purified according to the protocol using AMPure XP beads (Beckman Coulter, USA). Prior to amplicon sequencing, the amplicon pool was diluted in 10 mM Tris–HCl (pH 8.5) to a final concentration of 4 nM. Following the Illumina recommendations, the amplicon pool was denaturated using an equal amount of 0.2 M NaOH (BioUltra) (Sigma Aldrich Chemie GmbH, Germany) and further diluted to 12 pM in hybridization buffer (HT1 buffer included in the Reagents Kit v3, Illumina, USA). The pool was finally spiked with 5% denaturated PhiX control library (Illumina, USA). The sequencing was performed using the MiSeq sequencing platform with the Reagents Kit v3, 600 cycles (Illumina, USA).

### Analyses of 16S rRNA gene sequencing: composition, diversity, and discovery of metagenomic biomarkers

Sequence read data were demultiplexed using deML [47] before the composition and diversity of the gut microbiome were assessed using QIIME2 [48]. Initially, read-pairs were quality-filtered using q2-demux and denoised using DADA2 [49]. The total sequencing yield was 49,284,635 demultiplexed sequence counts, with a mean/median of 140,013/141,420 counts/sample. Reads were assigned to ASVs using q2-feature-classifier [50] for databases SILVA119 [51] and Greengenes [52] 13.8 (99% ASVs from 515F/806R region of sequences), with a total frequency of 13,501,064 and a mean frequency per sample of 39,020. The total number of features was 2585. For core diversity, both phylogenetic and non-phylogenetic metrics, we applied core-metrics-phylogenetic command with a sampling depth of 20,000. Within-sample diversity, i.e., α-diversity, was calculated using Faith’s phylogenetic diversity [53]. β-Diversity (pairwise sample dissimilarity) was calculated using unweighted UniFrac [54, 55] and is presented as principal coordinate analysis (PCoA). We used q2 qiime longitudinal maturity-index script [25, 26] to calculate the microbial maturity score (MAZ-score) longitudinally, between the intervention groups.

For the investigation of putative biomarkers between the groups, the LEfSe [22] pipeline (linear discriminant analysis (LDA) effect size) was used at the genus taxonomical level (L6 Greengenes). The LEfSe algorithm uses the non-parametric factorial Kruskal–Wallis sum rank test, unpaired Wilcoxon rank-sum test, and LDA, to estimate the effect size of each differentially abundant ASV and to pass significance—α parameter for tests was set to 0.05, and the threshold on the logarithmic score of LDA analysis was set to 2.0. Multivariable associations between the groups and taxonomic abundance were assessed using the *MaAsLin2* package [23]. Before analysis with *MaAsLin2*, taxa were agglomerated to the genus level. The compound Poisson linear model (CPLM) function was utilized on total sum scaled (TSS) normalized data and standardization of covariates to *z* scores. All *p*-values were false discovery rate-adjusted (Benjamini-Hochberg, *q*-values), and features with *q* < 0.25 were considered significant. ANCOM BC [24] was used to investigate the differential abundance longitudinally between the intervention groups at the genus (D__5 SILVA) and species (D__6 SILVA) taxonomical levels.

### Statistics

The Mann–Whitney *U* test or Test of Equal or Given Proportions was used to assess the differences in demographic characteristics. Statistical analyses and visualizations were performed using R 3.2.3 [56], SPSS version 25 (SPSS Inc., Chicago, IL, USA), and GraphPad Prism 8.4.3.

### Fecal metabolic profiling

One to 200 mg of freeze-dried feces samples was prepared as described in Jiye et al. [57] with some modifications. In brief, 1000 µL of extraction buffer (90/10 v/v methanol: water) including internal standards (see source data 2) was added to 5 mg (sample weights spanning from 4.02–6.98 mg) of freeze-dried feces. One tungsten bead was added to each sample, and the sample was shaken at 30 Hz for 3 min in a mixer mill. The tungsten bead was removed, and the samples were centrifuged at + 4 °C, 14,000 rpm, for 10 min. The supernatant, 75 µL for LCMS analysis and 25 µL for GCMS analysis, was transferred to microvials and evaporated to dryness in a speed-vac concentrator. Solvents were evaporated, and the samples were stored at − 80 °C until analysis. A small aliquot of the remaining supernatants was pooled and used to create quality control (QC) samples. MSMS analysis (LCMS) was run on the QC samples for identification purposes. The samples were analyzed in batches according to a randomized run order.

Derivatization and GCMS analysis were performed as described previously [58]. 0.5 μL of the derivatized sample was injected in splitless mode by a L-PAL3 autosampler (CTC Analytics AG, Switzerland) into an Agilent 7890B gas chromatograph equipped with a 10 m × 0.18 mm fused silica capillary column with a chemically bonded 0.18 μm Rxi-5 Sil MS stationary phase (Restek Corporation, USA). The injector temperature was 270 °C, the purge flow rate was 20 mL min^−1^, and the purge was turned on after 60 s. The gas flow rate through the column was 1 mL min^−1^, the column temperature was held at 70 °C for 2 min, then increased by 40 °C min^−1^ to 320 °C and held there for 2 min. The column effluent was introduced into the ion source of a Pegasus BT time-of-flight mass spectrometer, GC/TOFMS (Leco Corp., St Joseph, MI, USA). The transfer line and the ion source temperatures were 250 °C and 200 °C, respectively. Ions were generated by a 70-eV electron beam at an ionization current of 2.0 mA, and 30 spectra s^−1^ were recorded in the mass range m/z 50–800. The acceleration voltage was turned on after a solvent delay of 150 s. The detector voltage was 1800–2300 V. Before LCMS analysis, the sample was re-suspended in 10 + 10 µL methanol and water. Each batch of samples was first analyzed in positive mode. After all samples within a batch had been analyzed, the instrument was switched to negative mode and a second injection of each sample was performed. The chromatographic separation was performed on an Agilent 1290 Infinity UHPLC-system (Agilent Technologies, Waldbronn, Germany); 2 μL of each sample was injected onto an Acquity UPLC HSS T3, 2.1 × 50 mm, 1.8 μm C18 column in combination with a 2.1 mm × 5 mm, 1.8 μm VanGuard precolumn (Waters Corporation, Milford, MA, USA) held at 40 °C. The gradient elution buffers were A (H2O, 0.1% formic acid) and B (75/25 acetonitrile:2-propanol, 0.1% formic acid), and the flow rate was 0.5 mL min^−1^. The compounds were eluted with a linear gradient consisting of 0.1–10% B over 2 min, B was increased to 99% over 5 min and held at 99% for 2 min; B was decreased to 0.1% for 0.3 min, and the flow rate was increased to 0.8 mL min^−1^ for 0.5 min; these conditions were held for 0.9 min, after which the flow rate was reduced to 0.5 mL min^−1^ for 0.1 min before the next injection.

The compounds were detected with an Agilent 6550 Q-TOF mass spectrometer equipped with a jet stream electrospray ion source operating in positive or negative ion mode. The settings were kept identical between the modes, with the exception of the capillary voltage. A reference interface was connected for accurate mass measurements; the reference ion purine (4 μM) and HP-0921 (Hexakis(1H, 1H, 3H-tetrafluoropropoxy)phosphazine) (1 μM) were infused directly into the MS at a flow rate of 0.05 mL min^−1^ for internal calibration, and the monitored ions were purine m/z 121.05 and m/z 119.03632; HP-0921 m/z 922.0098 and m/z 966.000725 for positive and negative mode, respectively. The gas temperature was set to 150 °C, the drying gas flow to 16 L min^−1^, and the nebulizer pressure 35 psig. The sheath gas temp was set to 350 °C, and the sheath gas flow was 11 L min^−1^. The capillary voltage was set to 4000 V in positive ion mode and to 4000 V in negative ion mode. The nozzle voltage was 300 V. The fragmentor voltage was 380 V, the skimmer 45 V, and the OCT 1 RF Vpp 750 V. The collision energy was set to 0 V. The m/z range was 70–1700, and data were collected in centroid mode with an acquisition rate of 4 scans s-1 (1977 transients/spectrum).

### Analyses of fecal metabolites

For the GC–MS data, all non-processed MS files from the metabolic analysis were exported from the ChromaTOF software in NetCDF format to MATLAB R2016a (Mathworks, Natick, MA, USA), where all data pre-treatment procedures, such as base-line correction, chromatogram alignment, data compression, and multivariate curve resolution, were performed. The extracted mass spectra were identified by comparisons of their retention index and mass spectra with libraries of retention time indices and mass spectra [58]. Mass spectra and retention index comparison was performed using the NIST MS 2.0 software. Annotation of mass spectra was based on reverse and forward searches in the library. Masses and ratios between masses indicative of a derivatized metabolite were especially notified. If the mass spectrum according to SMC’s experience was with the highest probability indicative of a metabolite and the retention index between the sample and library for the suggested metabolite was ± 5 (usually less than 3), the deconvoluted “peak” was annotated as an identification of a metabolite. For the LC–MS data, all data processing was performed using the Agilent Masshunter Profinder version B.10.00 (Agilent Technologies Inc., Santa Clara, CA, USA). The processing was performed both in a target and an untargeted fashion. For target processing, a pre-defined list of metabolites commonly found in the plasma and serum was searched for using the Batch Targeted feature extraction in Masshunter Profinder. An in-house LC–MS library built up by authentic standards run on the same system with the same chromatographic and mass-spec settings was used for the targeted processing. The identification of the metabolites was based on MS, MSMS, and retention time information. The mixed-effects model was used to calculate the differences between the groups over time, and the Mann–Whitney *U* test was used to investigate if the metabolite was different between the groups at one, certain time point. Multivariate statistical investigations (PCA, OPLS-DA) were performed using pcaMethods [59] in R v3.2.3 [56].

### Preparation of the metagenomic sequencing library

Eleven infants with LRTI during the study period and 11 controls matched for the intervention group, age, sex, and weight (Fig. 1, Table 2) were selected for analyses of the gut metagenomic microbial community; 750 ng of clean and concentrated DNA (Genomic DNA clean & Concentrator™) (Cat#4064, The Epigenetics Company, USA) was used for library preparation using the TrueSeq Nano DNA library preparation kit (Illumina Inc.). Cluster generation and 150 cycles paired-end sequencing of sample libraries were performed in one S2-flowcell using the NovaSeq system and v1 sequencing chemistry (Illumina Inc.). Library preparation and shotgun sequencing were performed in collaboration with SNP&SEQ Technology Platform, Uppsala Biomedical Centre (BMC).

### Metagenomic analyses

Illumina paired-end reads were demultiplexed by index sequence before any downstream analysis. Adapter and index sequences were trimmed, and sequences were quality-filtered with Trimmomatic [60] before depletion of human contamination by removing all reads assigned to the human genome according to kraken2 [61] using a human-specific database. Sequence read assignments were performed using kraken2 based on a database downloaded from NCBI (March 9, 2020) consisting of the following sections: archea, bacteria, fungi, human, nt, protozoa, and viral. Taxonomic read assignments were done setting confidence to 0.1. Interactive analysis and visualization of kraken2 classifications were performed in Pavian [62]. ARIBA [63] was used to identify AMR-associated genes directly from individual sequence reads using CARD (v. 3.0.5). AMR-associated genes with less than 500 assigned reads were removed using R before being visualized using the package pheatmap. ATLAS [64] was used to assemble and characterize high-quality metagenome-assembled genomes (MAG) from the dataset, and the results were visualized using iTOL [65]. Analysis was performed using the workflow manager Snakemake [66] together with Bioconda [67] to allow efficient and automated deployment.

## Supplementary Information

**Additional file 1: Table S1.** Anthropometrical characteristics of subjects included for the fecal metabolome analyses. **Fig. S1.** Differences in microbial diversity and maturity between the intervention groups over time. **Fig. S2.** Metabolomics; time and intervention effects. **Fig. S3.** Abundance and distribution of *Bifidobacterium* species between the LRTI group (cases) and controls. **Fig. S4.** Abundance and distribution of *Klebsiella* species between the LRTI group (cases) and controls.

**Additional file 2.**

## Data Availability

The datasets supporting the conclusions in this article are available in SRA (amplicon and metagenomics sequences in the fastq format with limited metadata) under the bioproject number PRJNA656700 [68]. MAGs data protection legislation prohibits sharing of individual data, even when pseudonymized. Datasets regarding obtained metabolites are available in MetaboLights [69] under the study number MTBLS2438 [70]. The authors will share aggregate data that do not allow the identification of individuals, upon reasonable request. Correspondence and requests for material should be addressed to Dr. Christina E. West.
