# Supplementary material for: Targeting the gut-lung axis by synbiotic feeding to infants in a randomized controlled trial
Source: BMC Biol. 2023 Feb 20;21:38. doi: 10.1186/s12915-023-01531-3 (PMC9940374; doi:10.1186/s12915-023-01531-3)

Additional file 1

Targeting the gut-lung axis by synbiotic feeding to infants in a randomized controlled trial

Kotryna Simonyté Sjödin, Andreas Sjödin, Marek Ruszczyński, Mette Bach Kristensen, Olle Hernell, Hania Szajewska, Christina E West

Fig. S1. Comparison of microbial diversity and maturity between the prebiotic and synbiotic groups in the first year of life.

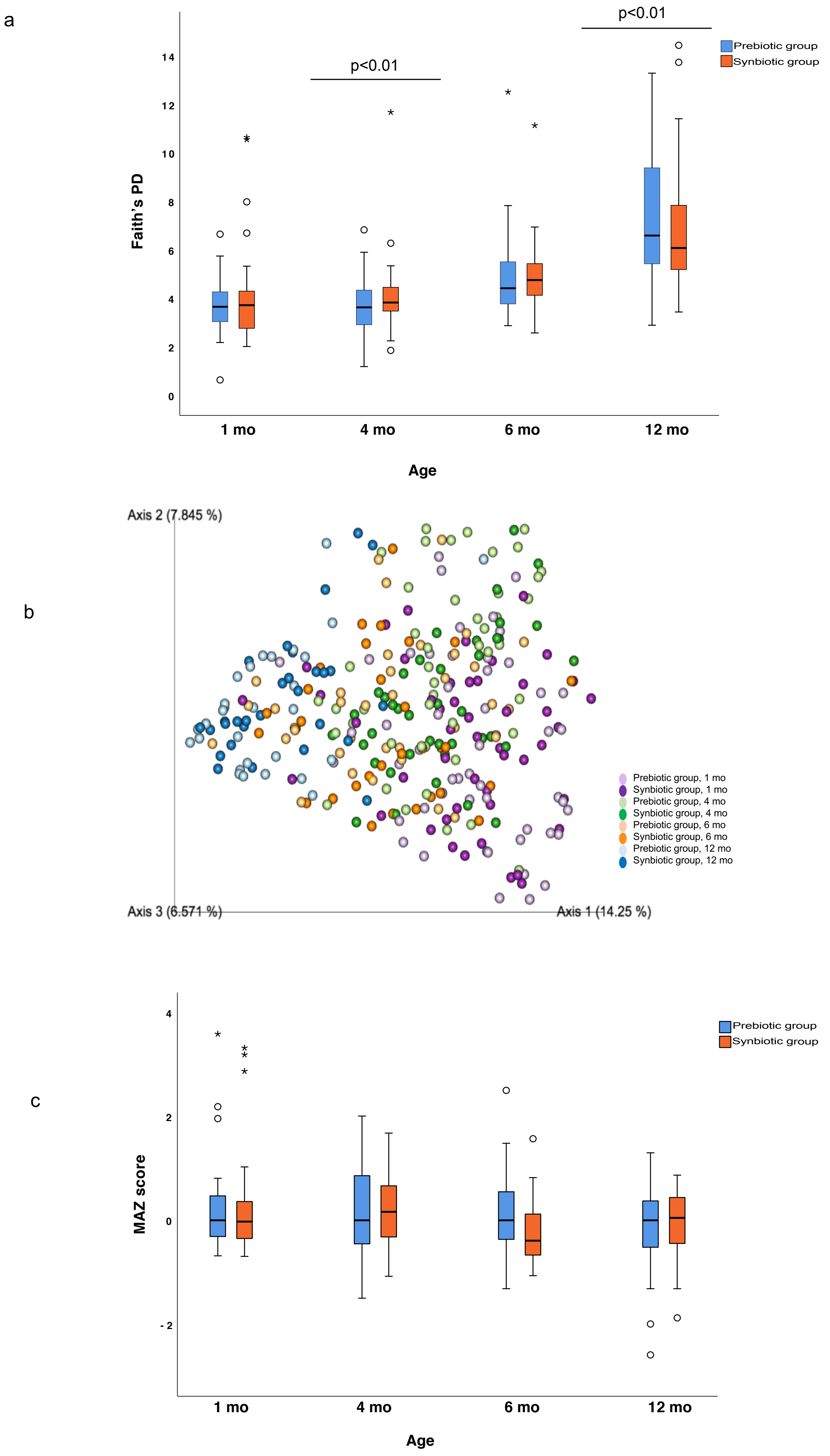

a) Box plots presenting Faith's PD as a measure of α-diversity in the prebiotic (blue) and synbiotic (orange) groups, ($p<0.01$, Kruskal-Wallis) b) PCoA plot presenting the microbial dissimilarity between the prebiotic and synbiotic groups in the first year of life ($p<0.001$, PERMANOVA, number of permutations 999) c) Box plots presenting the maturity-for-age Z (MAZ) score in the prebiotic and synbiotic groups. Prebiotic group (n=56 at 1 month, n=47 at 4 months, n=38 at 6 months and n=31 at 12 months). Synbiotic group (n=50 at 1 month, n=42 at 4 months, n=33 at 6 months and n=33 at 12 months).

Table S1. Demographic and anthropometrical characteristics of subjects included for analyses of the fecal metabolome

	Visit 1 (1 month of age)		Visit 2 (4 months of age)		Visit 3 (6 months of age)		Visit 4 (12 months of age)	
	**Prebiotics**	**Synbiotics**	**Prebiotics**	**Synbiotics**	**Prebiotics**	**Synbiotics**	**Prebiotics**	**Synbiotics**
**N (*)**	24	22	24	22	26	18	25	24
**Girls, %**	54	54	54	54	54	56	56	54
**Gestational age, weeks**	39 ± 1.1	40 ± 1.3	na	na	na	na	na	na
**Age, months**	0.7 ± 0.2	0.8 ± 0.2	3.9 ± 0.2	3.9 ± 0.1	5.9 ± 0.4	5.9 ± 0.1	12.1 ± 0.3	12.8 ± 0.2
**Height, cm**	55.4 ± 2.8	56.1 ± 2.5	65.8 ± 2.4	66.1 ± 2.3	70.1 ± 3.4	71.3 ± 4.0	80.7 ± 3.8	80.8 ± 3.7
**Weight, kg**	3.9 ± 0.4	4.1 ± 0.5	6.8 ± 0.7	7.1 ± 0.6	***7.9 ± 0.8***	***8.3 ± 0.6#***	10.2 ± 1.3	10.2 ± 0.9
**Head circumference, cm**	36.1 ± 1.4	36.4 ± 1.1	41.5 ± 1.4	41.5 ± 1.0	43.2 ± 1.3	43.4 ± 0.9	46.3 ± 2.1	46.1 ± 1.3
**BMI z-score**	-0.96± 0.8	-0.75± 0.8	-0.8± 0.8	-0.4± 1.0	-0.8± 1.1	-0.5± 1.4	-0.99± 1.6	-0.97± 1.5

(*) All subjects were vaginally delivered and predominantly formula-fed at inclusion
Data are presented as mean ± SD
(#) p=0.02, Mann-Whitney U test

Fig. S2. Metabolomics: time and intervention effects.

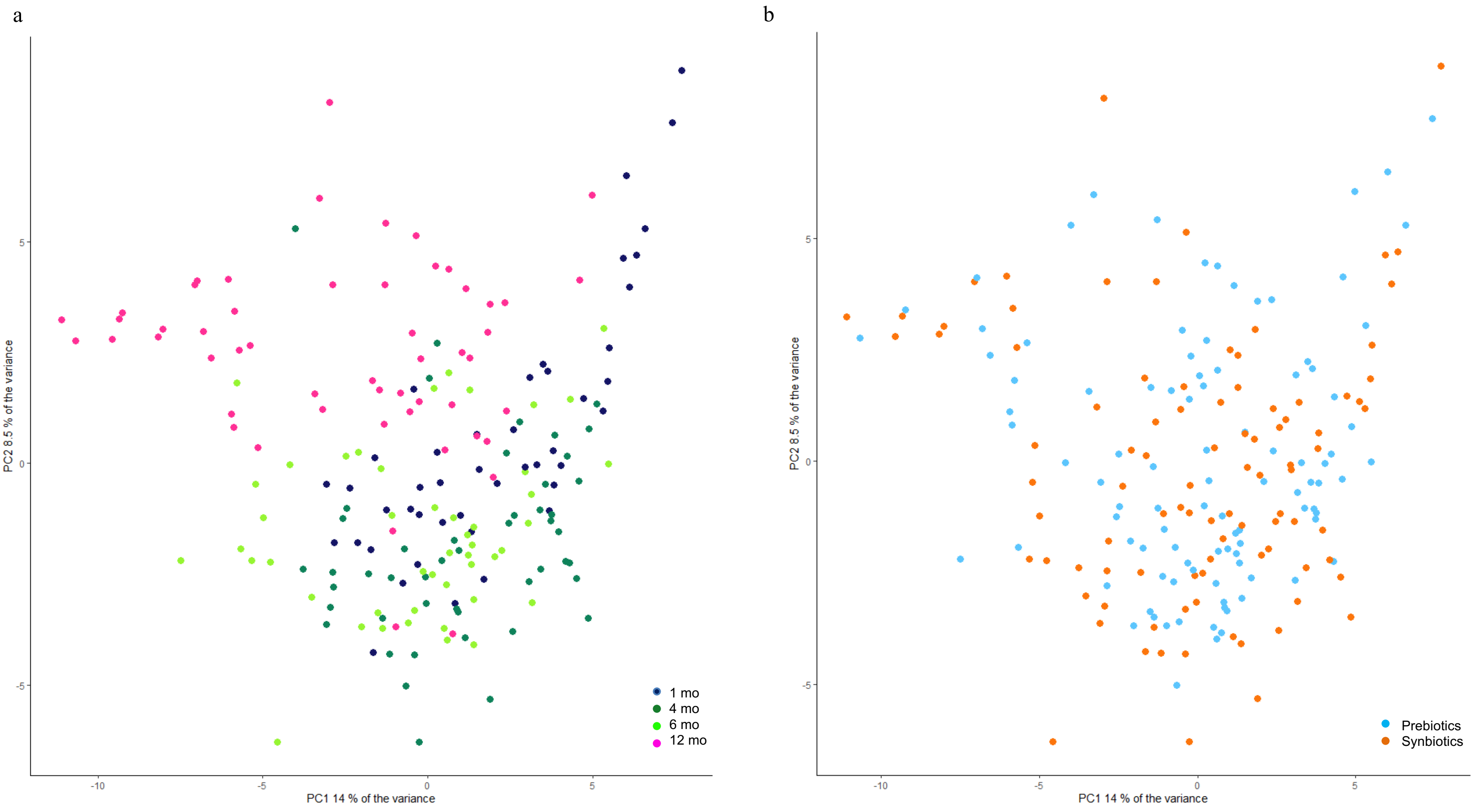

Time (a) and intervention (b) related differences in the fecal metabolic profile. a) Differences in the overall metabolite profile at 12 months (pink dots) compared with the earlier ages (p<0.001, paired Kruskal-Wallis test). b) Comparison of overall metabolite profile between the prebiotic (blue) and synbiotic (orange) groups. Prebiotic group (n=24 at 1 and 4 months, n=26 at 6 months and n=25 at 12 months). Synbiotic group (n=22 at 1 and 4 months, n=18 at 6 months and n=24 at 12 months). Source data are provided as a Source Data file.

Fig. S3. Abundance and distribution of *Bifidobacterium* species between LRTI group (cases; n=9) and controls (n=10)

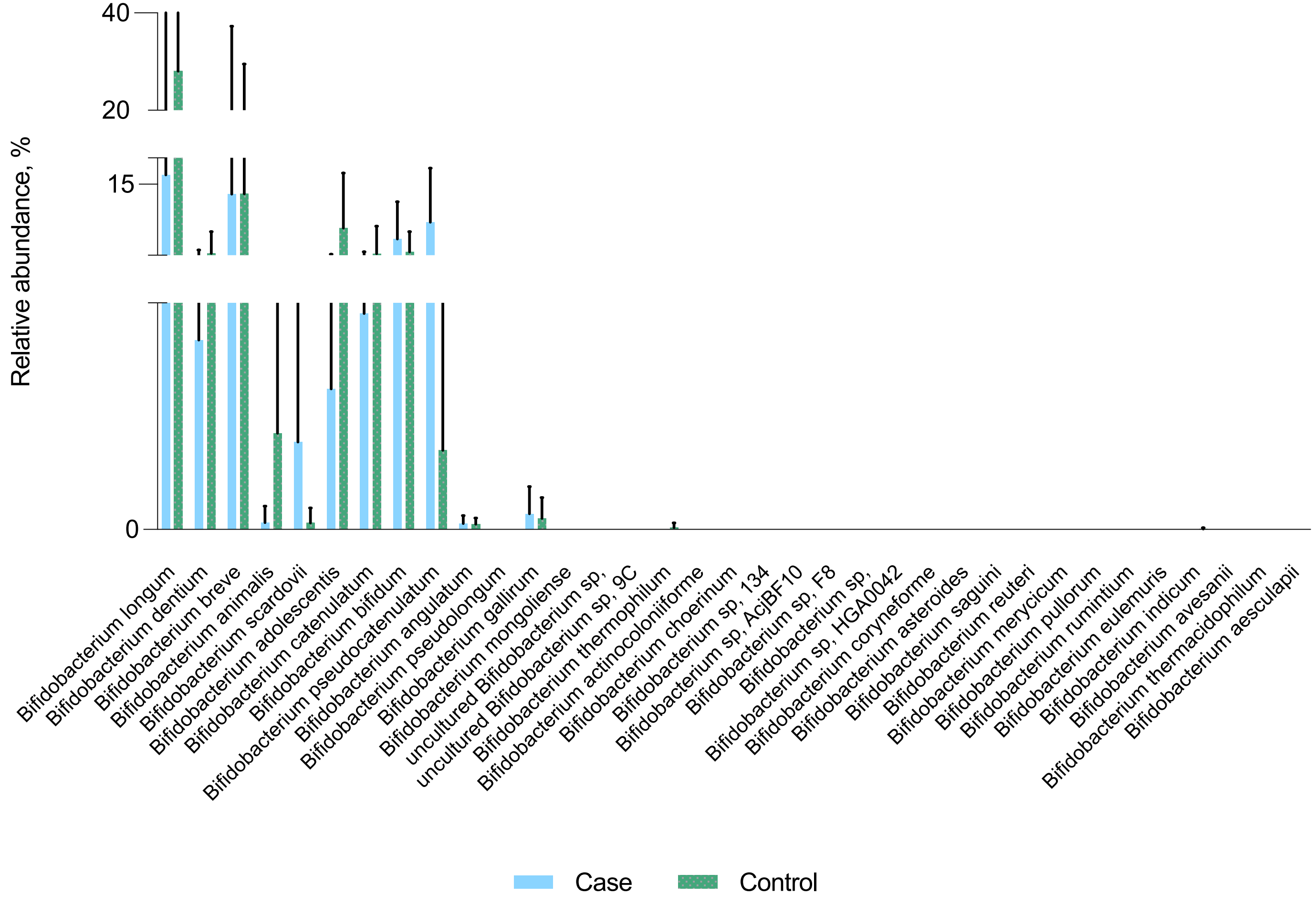

Fig. S4. Abundance and distribution of *Klebsiella* species between LRTI group (cases; n=9) and controls (n=10)

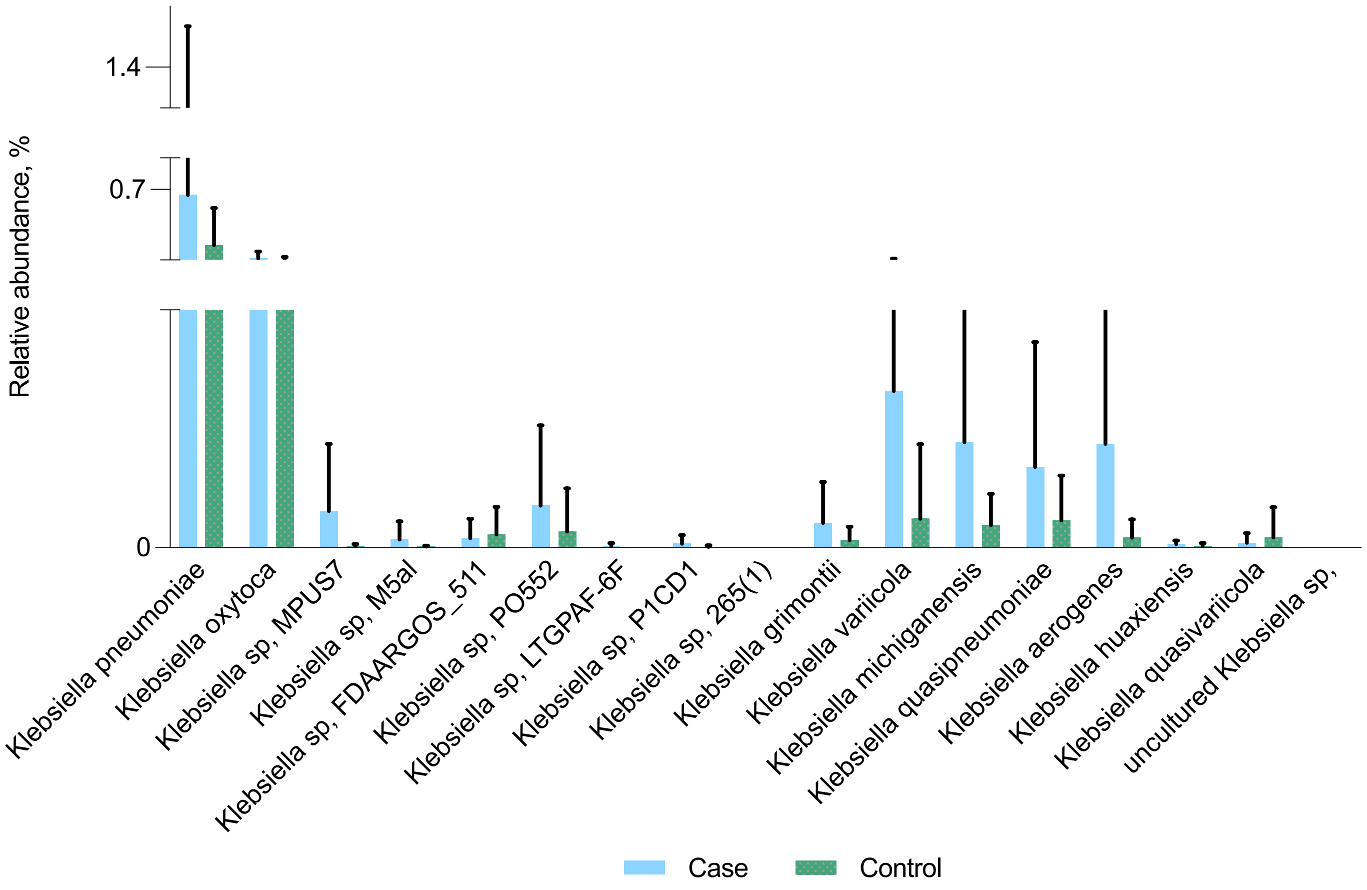

Supplement: Supplementary file 1 — Additional file 1: Table S1. Anthropometrical characteristics of subjects included for the fecal metabolome analyses. Fig. S1. Differences in microbial diversity and maturity between the intervention groups over time. Fig. S2. Metabolomics; time and intervention effects. Fig. S3. Abundance and distribution of Bifidobacterium species between the LRTI group (cases) and controls. Fig. S4. Abundance and distribution of Klebsiella species between the LRTI group (cases) and controls. [file 12915_2023_1531_MOESM1_ESM.pdf]
